# Supplementary material for: Improving Cohort-Hospital Matching Accuracy through Standardization and Validation of Participant Identifiable Information
Source: Children (Basel). 2022 Dec 7;9(12):1916. doi: 10.3390/children9121916 (PMC9776599; doi:10.3390/children9121916)
Supplement: Supplementary file 1 [file children-09-01916-s001.zip › children-2001796-supplementary.pdf]

children

Article

Improving Cohort-Hospital Matching Accuracy through Standardization and Validation of Participant Identifiable Information

Yanhong Jessika Hu [1,2], Anna Fedyukova [1], Jing Wang [1,2], Joanne M. Said [1,3,4], Niranjan Thomas [3,4], Elizabeth Noble [4], Jeanie L. Y. Cheong [1,3,5], Bill Karanatsios [6], Sharon Goldfeld [1,7] and Melissa Wake [1,2,*]

1 Murdoch Children's Research Institute, The Royal Children's Hospital, Parkville, VIC 3052, Australia
2 Department of Pediatrics, The University of Melbourne, Parkville, VIC 3052, Australia
3 Department of Obstetrics and Gynaecology, The University of Melbourne, Parkville, VIC 3010, Australia
4 Maternal Fetal Medicine, Joan Kirner Women's & Children's at Sunshine Hospital, St Albans, VIC 3021, Australia
5 Newborn Research, The Royal Women's Hospital, Parkville, VIC 3052, Australia
6 Western Health Chronic Disease Alliance, Western Health, St Albans, VIC 3021, Australia
7 Centre for Community Child Health, The Royal Children's Hospital, Parkville, VIC 3052, Australia
* Correspondence: melissa.wake@mcri.edu.au

Citation: Hu, Y.J.; Fedyukova, A.; Wang, J.; Said, J.M.; Thomas, N.; Noble, E.; Cheong, J.L.Y.; Karanatsios, B.; Goldfeld, S.; Wake, M. Improving Cohort-Hospital Matching Accuracy through Standardization and Validation of Participant Identifiable Information. *Children* **2022**, *9*, 1916. https://doi.org/10.3390/children9121916

Academic Editor: Henry C. Lee

Received: 15 October 2022
Accepted: 3 December 2022
Published: 7 December 2022

Publisher's Note: MDPI stays neutral with regard to jurisdictional claims in published maps and institutional affiliations.

Copyright: © 2022 by the authors. Licensee MDPI, Basel, Switzerland. This article is an open access article distributed under the terms and conditions of the Creative Commons Attribution (CC BY) license (https://creativecommons.org/licenses/by/4.0/).

Supplementary Materials:

Supplementary Tables

Table S1. Mapping dictionary for standardization of address suffixes.

GenV dataset address suffixes	Hospital dataset address suffixes
Court	Ct
Road	Rd
Avenue	Ave
Street	St
Drive	Dr
Boulevard	Bvd
Crescent	Cres
Close	Cl
Quadrant	Qdrt
Place	Pl
Parade	Pde
Grove	Gr
Boulevard	Bvd
Circuit	Cct
Gardens	Gdns
Square	Sq
Terrace	Tce
Oconnor	O'Connor

green fields	Greenfields

Note: Phone number and address are mock-up examples.

Table S2. Examples of address mismatching reasons requiring manual check.

GenV	Hospital
U 5 20 University Ct	U 6 20 University Ct
90 Northlake	91 Northlake Ave
10 green fields boulevard	10 Greenfields Bvd
U 114 12 Country Rd	U 114 12 Bread St
5 Holwell Ave	5 Howell Ave
7 Eastville St	7 Eastfield St
27 Oneill Rd	27 O'Neill Rd
15 Illinabee Dr	15 Allinabee Dr

Note: All addresses were mocked up from the datasets to illustrate the same issues.

Table S3. Hospital returned the variable list for GenV participants for further matching analysis.

Name of the variables from the Birthing Hospital	Mother or baby	Comment
**For Scenario 1 and Scenario 2:**		
ID	Both	new ID, generated by computer and different from any used in the internal GenV systems.
IP Episode ID	Baby	Episode ID for a baby
B-DOB BOS full		Baby Birthdate (DOB) with time
B-DOB BOS		Baby Birthdate (DOB) without time
Patient Given Name		Baby First Name (FN)
Patient Surname		Baby Last Name (LN)
Gender		Baby Gender (Sex)
Baby birth order Number		Birth Order (BO) for multiple births (e.g. twins, triplets)
Plurality		Singleton/ Twins
Episode Number	Biological Mother	Episode ID for a mother
M-Given name iPM	Biological Mother	Biological Mother’s First Name (FN)
M-Surname iPM		Biological Mother’s Last Name (LN)
M-DOB iPM		Biological Mother’s DOB
M-DOB dd/mm iPM		Day and month of mother’s DOB
M-DOB birth year iPM		Year of mother’s DOB
Address		Mother’s Street Address (ADD)
iPM mobile number		Mother’s Phone number (TN)

iPM other phone		Mother's other Phone number (TN)
**For Scenario 3:**		
ID	Both	new ID, generated by computer and different from any used in the internal GenV systems.
IP Episode ID	Baby	Episode ID for a baby
B-DOB BOS full	Baby	Baby Birthdate (DOB) without time
B-DOB iPM		Baby Birthdate (DOB) with time
Patient Given Name		Baby First Name (FN)
Patient Surname		Baby Last Name (LN)
Baby Number		Birth Order (BO) for multiple births
Baby Gender (Sex)		Baby Gender (Sex)
Plurality		Single/multiple (e.g. twins, triplets)
Address	Other parent	other parent's Street Address (ADD)
iPM phone number		other parent's Phone number (TN)

Table S4. Reporting guidelines for studies using data linkage.

DOMAIN 1: The existing data sources to be linked (complete for each dataset to be linked in the study)		
	Dataset1 (GenV cohort)	Dataset2 (Birthing hospital)
1. Purpose of the dataset was given	✓	✓
2. Description of the type of dataset (administrative/ clinical registry/ research study) was specified	✓	✓
3. Any standardised coding system/data dictionary used should was stated	✓	✓
4. % population coverage		✓
5. Data collection methods were described	✓	✓
6. Data quality assurance process described, including the frequency of checks	✓	✓
DOMAIN 2: Researcher-selected variables and data preparation		
7. Were the participant inclusion criteria specified?		✓
8. Were the variables used for linkage stated (including rates of missing data)?		✓
9. Were changes to the coding systems reported (including changes over time or revisions to disease/risk factor definitions revised during the study period)?		Not applicable
10. Were potential sources of bias adequately described?		Not applicable
DOMAIN 3: Technology/linkage process		
11. Was the intended precision of the linkage stated?		✓
12. Was a description of the linkage method given (e.g. deterministic, probabilistic methods including use of blocking and phonetic coding, if used) and a justification for the use of this type of linkage provided?		✓

13.Was a measure of the quality of the linked data-sets provided (e.g. % linked records, false positive/false negative rates)?	✓ (accuracy rate defined)
14. Did the study receive approval from a human research ethics committee?	✓

Table S5. The RECORD statement – checklist of items, extended from the STROBE statement, that should be reported in observational studies using routinely collected health data.

**Domain**	**Item No.**	**RECORD items**	**Check**
	1	RECORD 1.1: The type of data used should be specified in the title or abstract. When possible, the name of the databases used should be included.<br>RECORD 1.2: If applicable, the geographic region and timeframe within which the study took place should be reported in the title or abstract.<br>RECORD 1.3: If linkage between databases was conducted for the study, this should be clearly stated in the title or abstract.	✓
Background rationale	2	Explain the scientific background and rationale for the investigation being reported	✓
Objectives	3	State specific objectives, including any prespecified hypotheses	✓
Study Design	4	Present key elements of study design early in the paper	✓
Setting	5	Describe the setting, locations, and relevant dates, including periods of recruitment, exposure, follow-up, and data collection	✓<br>Exposure, follow-up is not applicable in this report
Participants	6	RECORD 6.1: The methods of study population selection (such as codes or algorithms used to identify subjects) should be listed in detail. If this is not possible, an explanation should be provided.<br>RECORD 6.2: Any validation studies of the codes or algorithms used to select the population should be referenced. If validation was conducted for this study and not published elsewhere, detailed methods and results should be provided.<br>RECORD 6.3: If the study involved linkage of databases, consider use of a flow diagram or other graphical display to demonstrate the data linkage process, including the number of individuals with linked data at each stage.<br>Cohort study - For matched studies, give matching criteria and number of exposed and unexposed	✓
Variables	7	RECORD 7.1: A complete list of codes and algorithms used to classify exposures, outcomes, confounders, and effect modifiers should be provided. If these cannot be reported, an explanation should be provided.	✓
Data sources/ measurement	8	For each variable of interest, give sources of data and details of methods of assessment (measurement).<br>Describe comparability of assessment methods if there is more than one group	✓
Bias	9	Describe any efforts to address potential sources of bias	Not applicable
Study size	10	Explain how the study size was arrived at	✓

Quantitative variables	11	Explain how quantitative variables were handled in the analyses. If applicable, describe which groupings were chosen, and why	✓
Statistical methods	12	(a)Describe all statistical methods, including those used to control for confounding<br>(b) Describe any methods used to examine subgroups and interactions<br>(c) Explain how missing data were addressed	Not applicable
Data access and cleaning methods		RECORD 12.1: Authors should describe the extent to which the investigators had access to the database population used to create the study population.<br>RECORD 12.2: Authors should provide information on the data cleaning methods used in the study.	✓
Linkage		RECORD 12.3: State whether the study included person-level, institutional-level, or other data linkage across two or more databases. The methods of linkage and methods of linkage quality evaluation should be provided.	✓
Participants	13	RECORD 13.1: Describe in detail the selection of the persons included in the study (i.e., study population selection) including filtering based on data quality, data availability and linkage. The selection of included persons can be described in the text and/or by means of the study flow diagram.	✓
Descriptive data	14	(a) Give characteristics of study participants (e.g., demographic, clinical, social) and information on exposures and potential confounders<br>(b) Indicate the number of participants with missing data for each variable of interest	Only demographic data involved in this study
Key results	18	Summarise key results with reference to study objectives	✓
Limitations	19	RECORD 19.1: Discuss the implications of using data that were not created or collected to answer the specific research question(s). Include discussion of misclassification bias, unmeasured confounding, missing data, and changing eligibility over time, as they pertain to the study being reported.	✓
Interpretation	20	Give a cautious overall interpretation of results considering objectives, limitations, multiplicity of analyses, results from similar studies, and other relevant evidence.	✓
Generalisability	21	Discuss the generalisability (external validity) of the study results	✓
Funding	22	Give the source of funding and the role of the funders for the present study and, if applicable, for the original study on which the present article is based	✓
Accessibility of protocol, raw data, and programming code		RECORD 22.1: Authors should provide information on how to access any supplemental information such as the study protocol, raw data, or programming code.	✓

Table S6. Summary of the three matching approaches and their interactions steps with different variables.

	**Deterministic rule-based approach (Mother and baby variables)**							**SLK-5881 approach (2nd, 3rd letters of M-FN, 2nd, 3rd, and 5th letters of M-LN, M-DOB, B-DOB, B-BO)**							**SLK-5881.1 approach (first 2 letter of M-FN, first 3 letters of M-LN, M-DOB, B-DOB, B-BO)**						
	M-FN	M-LN	M-DOB	M-ADD	M-TN	B-DOB	BO	M-FN	M-LN	M-DOB	B-DOB	BO	M-TN		M-FN	M-LN	M-DOB	B-DOB	BO	M-TN	
**Mother-baby pairs**																					
Step 1	x	x	x	x	x	x	x	x	x	x	x	x			x	x	x	x	x		
Step 2	x	x	x		x	x	x	x	x		x	x	x		x	x		x	x	x	
Step 3		x	x	x	x	x	x	x		x	x	x	x		x		x	x	x	x	
Step 4	x	x	x	x		x	x														
Step 5	x	x		x		x	x														
Step 6	x		x	x	x	x	x														
Step 7			x		x	x	x														
Step 8		x		x		x	x														
Step 9		x			x	x	x														
Step 10	x	x	x			x	x														
**Baby without Mothers' PII**																					
	B-FN	B-LN	B-DOB	B-SEX	P2-TN	P2-ADD	BO	B-FN	B-LN	B-DOB	B-SEX	P2-TN	P2-ADD	BO	B-FN	B-LN	B-DOB	B-SEX	P2-TN	P2-ADD	BO
Step 1	x	x	x	x	x	x	x	x	x	x	x			x	x	x	x	x			x
Step 2		x	x	x		x	x		x	x	x	x		x		x	x	x	x		x
Step 3	x	x	x	x			x		x	x	x		x	x		x	x	x		x	x

Note: FN=first name; LN=last name; M-DOB=mother's date of birth; B-DOB=baby's date of birth; M-ADD= mother's address. BO=birth order; M-TN=mother telephone; P2-TN=the other parent's telephone; P2-ADD= the other parent's address.

Supplemental Figures

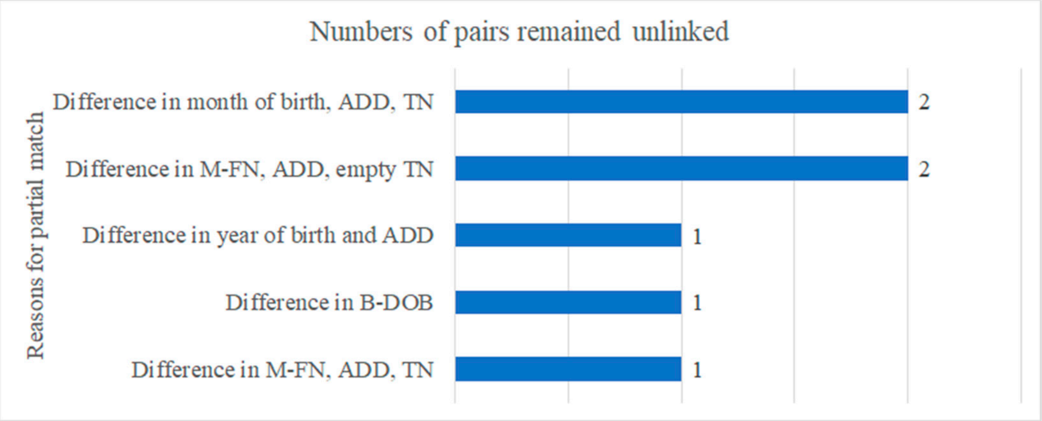

Figure S1. The reasons for partial matching for mother-baby pairs through deterministic rule-based approach.

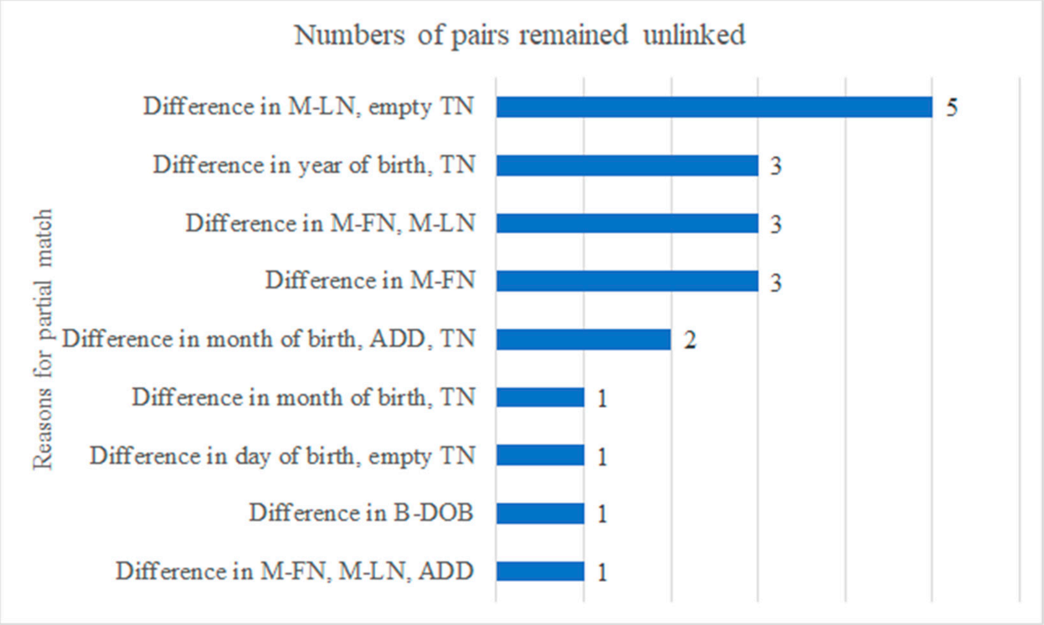

Figure S2. The reasons for partial matching for mother-baby pairs through SLK-5881 and SLK-5881.1 approaches.
